# Supplementary material for: Conceptualising emotional and cognitive dysregulation amongst sports bettors; an exploratory study of ‘tilting’ in a new context
Source: PLoS One. 2022 Feb 17;17(2):e0264000. doi: 10.1371/journal.pone.0264000 (PMC8853566; doi:10.1371/journal.pone.0264000)
Supplement: Attachment — Submitted filename: Response to Reviewers.docx [file pone.0264000.s002.docx]

Dear Editor,

Thank you very much for the review of our manuscript entitled: “Conceptualising emotional and cognitive dysregulation amongst sports bettors; an exploratory study of ‘tilting’ in a new context”. We sincerely appreciate all valuable comments and suggestions from you and the reviewer, which helped us to improve the quality of the article. Our responses to the comments are presented in the table on the following page. All of the necessary changes, suggested by you and the reviewer are highlighted within the document titled “Revised Manuscript with Track Changes”.

The co-authors and I hereby affirm that this manuscript has not been published previously, accepted for publication elsewhere and it is not under consideration for publication elsewhere.

We hope that our manuscript will now be acceptable for publication in PLOS ONE following your helpful comments.

Yours sincerely,

Jamie Torrance (corresponding author)

jamie.torrance@southwales.ac.uk

|  | **Editor and reviewer comments** | **Authors’ responses** |
| --- | --- | --- |
| **Editor** | Please ensure that your manuscript meets PLOS ONE's style requirements, including those for file naming. | **Title page:**  Title, authors, affiliations, and contributions have all been adjusted in line with the formatting requirements of PLOS ONE.  **Manuscript:**  “Abstract”, “Introduction”, “Methods”, “Results”, “Discussion”, “Conclusion”, “References” and “Supporting information” first-level titles changed to 18pt font and bold.  All second-level headings changed to 16pt font and bold.  All third-level headings changed to 14pt font and bold.  “Figure 1” changed to “Fig 1” (page 8 – line 217) and Fig 1 title changed to bold (page 9 – line 221).  “Figure 2” changed to “Fig 2” (page 12 – line 325) and Fig 2 title changed to bold (page 13 – line 331)  Table 1, 2, 3 and 4 titles have been changed to bold.  **Files:**  All file names have been corrected and adhere to PLOS ONE requirements.  Fig 1 and 2 were also uploaded to the Preflight Analysis and Conversion Engine (PACE) digital diagnostic tool and subsequently downloaded before being attached to this resubmission. |
|  | Please review your reference list to ensure that it is complete and correct. | The following references have been corrected and/or completed: 1, 4, 7, 8, 10, 21, 22, 23, 26, 28, 30, 31, 32, 34, 35, 39, 47, 49, 50, 52, 56  They are also highlighted in the reference list of the manuscript |
| **Reviewer 1** | On page 5 (line 103): Please correct “Titling” | This how now been corrected to “Tilting” (page 5 – line 99) |
|  | On page 6 (line 139): Please double check 20.44 there and 20.22 in table 1 and ensure consistent and correct information be reported in these places. | The value has been corrected and now matches the value displayed in Table 1. It now reads “with the largest proportion of participants (22.22%) representing the 18-24 age bracket (*n* = 50)” (page 6 – line 134) |
|  | On page 6 (line 146): Please double check 20.67 there – would this need to be revised to 51 based on table 2? | This has now been changed to reflect the data displayed in Table 2. It now reads “22.67% gambled a few times a week (*n* = 51)” (page 6 – line 141) |
|  | On page 8 (line 208, 210-211): “in-play betting product feature scale” was mentioned there as well as in line 290 on page 11, but Fig. 1 title mentions “in-play product preferences scale” – please try to use consistent wording throughout the paper. | Fig 1 title has been adjusted for consistency. It is now displayed as “Fig 1. Screenshot examples of the in-play betting product feature scale” (page 9 – line 221).  For clarity, the following adjustment was also made to the description of the scale to highlight that it measures preference. It now reads “As indicated within Fig 1, each product feature was presented alongside a brief description, a picture, and two preference-related questions measured via 5-point Likert scales” (page 8 – line 218). |
|  | On page 9 (line 248, 249): “Nine Item Online Poker Tilt Scale” and “The 9-item Online Poker Tilting Scale” were mentioned there – please try to use consistent wording throughout the paper. | The title of the scale has now been changed to “9-item Online Poker Tilting Scale (OPTS-9)” and is consistent with the line below (page 9 – line 244, 245). |
|  | On page 10 (line 254): “including” might need revision. | This has now been corrected. It reads “The key aspects of tilting that are covered by the items within the OPTS-9 include risk-taking, alteration of focus....” (page 10, line 250). |
|  | On page 14 (line 366): one of the two “that” needs to be deleted. | The second “that” has been deleted. The sentence now reads as follows: “The results of this test indicated that there was a statistically significant difference in gambling frequency between at least one pair of clusters” (page 14 – line 364). |
|  | On page 16 (line 409): first “between” needs to be revised to better wording. | This sentence has been adjusted for clarity. It now reads “Overall, the preferences differed significantly across some but not all of the in-play product features between the three clusters. (page 16 – line 408-409). |
|  | On page 20 (line 517-518): The sentence “However … groups.” needs to be revised. | This sentence has been revised for clarity. It now reads “However, there was no significant association between cluster membership and sports betting type (in-play or conventional)” (page 20 – line 516-517). |
|  | Some of the references in the reference list might have incomplete or incorrect information, or parts that need revision (e.g., reference 4, 47). Please double check all references and make sure their information is correct. | The following references have been corrected and/or completed: 1, 4, 7, 8, 10, 21, 22, 23, 26, 28, 30, 31, 32, 34, 35, 39, 47, 49, 50, 52, 56  They are also highlighted in the reference list of the manuscript |
